# Association of pre-existing comorbidities with outcome of allogeneic hematopoietic cell transplantation. A retrospective analysis from the EBMT

**DOI:** 10.1038/s41409-021-01502-8

**Published:** 2021-10-30

**Authors:** Olaf Penack, Christophe Peczynski, Mohamad Mohty, Ibrahim Yakoub-Agha, Rafael de la Camara, Bertram Glass, Rafael F. Duarte, Nicolaus Kröger, Hélène Schoemans, Christian Koenecke, Zinaida Peric, Grzegorz W. Basak

**Affiliations:** 1 Medical Clinic, Department of Haematology, Oncology and Tumor Immunology, Charité Universitätsmedizin Berlin, Berlin, Germany; 2 EBMT Transplant Complications Working Party, Paris, France; 3 Sorbonne University, Department of Haematology, Saint Antoine Hospital; INSERM UMR-S 938, Paris, France; 4 Department of Hematology, Hôpital Saint-Antoine, Universite Pierre & Marie Curie, INSERM UMR-S 938, Paris, France; 5 EBMT Acute Leukemia Working Party, Paris, France; 6 Univ Lille, Inserm, CHU Lille, INSERM, Infinite, U1286, F-59000 Lille, France; 7 EBMT Chronic Malignancies Working Party, Paris, France; 8 Hematology División, Hospital de la Princesa, Madrid, Spain; 9 EBMT Infectious Diseases Working Party, Paris, France; 10 Department of Hematology, Oncology, and Tumor Immunology, Helios Klinikum Berlin-Buch, Berlin, Germany; 11 EBMT Lymphoma Working Party, Paris, France; 12 Hematopoietic Transplantation and Hemato-Oncology Section, Hospital Universitario Puerta de Hierro Majadahonda, Madrid, Spain; 13 University Hospital Eppendorf, Hamburg, Germany; 14 Department of Hematology, University Hospitals Leuven and KU Leuven, Leuven, Belgium; 15 Department of Hematology, Hemostasis, Oncology and Stem Cell Transplantation, Hannover Medical School, Hannover, Germany; 16 Department of Hematology, University Hospital Centre Zagreb, Zagreb, Croatia; 17 Department of Hematology, Transplantation and Internal Medicine, Medical University of Warsaw, Warsaw, Poland

**Keywords:** Translational research, Risk factors

## Abstract

Risk assessment of allogeneic hematopoietic cell transplantation (allo-HCT) is hindered by the lack of current data on comorbidities and outcome. The EBMT identified 38,760 allo-HCT recipients with hematologic malignancies transplanted between 2010 and 2018 from matched sibling and unrelated donors with a full data set of pre-existing comorbidities. Multivariate analyses using the Cox proportional-hazards model including known risk factors for non-relapse mortality (NRM) were performed. We found that pre-existing renal comorbidity had the strongest association with NRM (hazard ratio [HR] 1.85 [95% CI 1.55–2.19]). In addition, the association of multiple pre-existing comorbidities with NRM was significant, including diabetes, infections, cardiac comorbidity, and pulmonary comorbidity. However, the HR of the association of these comorbidities with NRM was relatively low and did not exceed 1.24. Consequently, the risk of NRM was only moderately increased in patients with a high hematopoietic cell transplantation comorbidity index (HCT-CI) ≥ 3 (HR 1.34 [1.26–1.42]). In the current EBMT population, pre-existing non-renal comorbidities determined NRM after allo-HCT to a much lesser extent as compared with the underlying HCT-CI data. Improvements in management and supportive care as well as higher awareness based on the use of HCT-CI may have contributed to this favorable development.

## Introduction

Allogeneic hematopoietic cell transplantation (allo-HCT) is a curative treatment option for patients suffering from leukemia and other hematological malignancies. The use of allo-HCT is constantly increasing with nearly 20,000 transplantations reported to the European Society for Blood and Marrow Transplantation (EBMT) per year [1]. The main clinical challenge of allo-HCT, beyond relapse, is the high treatment-associated mortality, which is mainly driven by graft-versus-host disease (GVHD), infectious complications, and conditioning-related toxicities. For better individual assessment of allo-HCT risk, considerable progress has been made by defining comorbidities being associated with mortality. This led to the adaptation of the Charlson comorbidity index to the allo-HCT setting [2], creating the the Hematopoietic Cell Transplantation Comorbidity Index (HCT-CI) in 2005 [3, 4], which has proved to be of enormous clinical value to predict NRM. However, as allo-HCT continues to improve, with better donor choices and supportive care, the predictive value of HCT-CI needs to be frequently re-evaluated in the light of new practice patterns. To address this knowledge gap, the EBMT collects data on pre-existing comorbidities, transplant characteristics, and outcome. Here we present the first comprehensive EBMT database analysis on the association of comorbidities with outcome. We hypothesized that the association of pre-existing comorbidities with non-relapse mortality (NRM) after allo-HCT would be lower now as compared with the original HCT-CI population [3, 4]. The basis for our hypothesis was the improved overall NRM after alloSCT over time [5], as well as our assumption that the better awareness of comorbidities on the mortality risk had beneficial effects. We sought to validate this hypothesis in the EBMT database.

## Patients and methods

### Study design and data collection

This is a retrospective multicenter analysis using the data set of the EBMT registry. The EBMT is a voluntary working group of more than 600 transplant centers that are required to report regular follow-up on all consecutive stem cell transplantations. Audits are routinely performed to determine the accuracy of the data. The study was planned and approved by the Transplant Complications Working Party of the EBMT. All patients gave their written informed consent to use their personal information for research purposes. The study was conducted in accordance with the Declaration of Helsinki and Good Clinical Practice guidelines. Eligibility criteria for this analysis included patients older than 18 years of age at allo-HCT with hematologic malignancies including acute leukemia, chronic leukemia, lymphoma, myelodysplastic syndrome, or myeloproliferative neoplasms, who underwent a first allo-HCT (previous autologous transplantation(s) allowed) from a matched sibling donor or unrelated donor, using either bone marrow or peripheral blood, between 2010 and 2018. We only included patients with a full available data set on pre-existing comorbidities. Further exclusion criteria were lack of information on disease progression (status or date), conditioning (intensity, or use of total body irradiation), status at transplant. Data collected included recipient and donor characteristics (age, sex, cytomegalovirus (CMV) serostatus, and Karnofsky performance status (KPS) score), diagnosis and status at transplant, and transplant-related factors, including conditioning regimen, use of anti-thymocyte globulin or alemtuzumab for pre-transplant in vivo T-cell depletion, stem cell source, and post-transplant GVHD prophylaxis. Grading of acute GVHD was performed using established criteria [6]. Chronic GVHD was classified as limited or extensive, according to published criteria [7]. For the purpose of this study, all necessary data were collected according to the EBMT guidelines, using the EBMT Minimum Essential Data (Med) forms.

### Assessment and definition of comorbidities

At the beginning of 2016 an assessment of pre-existing comorbidities was added to the Med-A form, which is mandatory to submit to the EBMT for all alloSCT transplants in all EBMT centers. Assessment of comorbidities on the Med-A form included:

- Solid tumor treated at any time point in the patient’s past history; excluding non-melanoma skin cancer.
- Inflammatory bowel disease—Crohn’s disease or ulcerative colitis.
- Rheumatologic disease—Systemic lupus erythematosus, rheumatoid arthritis, polymyositis, mixed connective tissue disease, or polymyalgia rheumatica.
- Infection requiring continuation of antimicrobial treatment after day 0
- Diabetes requiring treatment with insulin or oral hypoglycemic drugs but not diet alone.
- Renal disease, moderate/severe—serum creatinine > 2 mg/dL or >177 mol/L, on dialysis, or prior renal transplantation.
- Hepatic disease—chronic hepatitis or bilirubin between upper limit of normal (ULN) and 1.5 x the ULN, or AST/ALT between ULN and 2.5 × ULN.
- Moderate/severe hepatic comorbidity—liver cirrhosis, bilirubin greater than 1.5 × ULN, or AST/ALT greater than 2.5 × ULN.
- Moderate pulmonary comorbidity—diffusing capacity of the lungs for carbon monoxide (DLCO) or forced expiratory volume in 1 s (FEV1) of 66–80% or dyspnea on slight activity.
- Severe pulmonary comorbidity —DLCO or FEV1 ≤ 65% or dyspnea at rest or requiring oxygen.
- Arrhythmia—atrial fibrillation or flutter, sick sinus syndrome, or ventricular arrhythmia.
- Cardiac disease—coronary artery disease, congestive heart failure, myocardial infarction, ejection fraction < 50%.
- Cerebrovascular disease—transient ischemic attack or cerebrovascular accident.
- Heart valve disease—all except mitral valve prolapse.
- Obesity—patients with a body mass index > 35 kg/m^2^.
- Peptic ulcer requiring treatment.
- Psychiatric disturbance—depression or anxiety requiring psychiatric consultation or treatment.

Before 2016, the documentation of comorbidities was not mandatory but was performed for a considerable number of alloSCTs. Patients without a full data set of comorbidities were excluded from analyses. We also excluded the patients with missing pulmonary severity data (around 1% and the patients), because these data are necessary to calculate the HCT-CI index (Sorror).

### Statistical analysis

The primary study endpoint was NRM. Secondary study endpoints were overall survival (OS), progression-free survival (PFS), cumulative incidence of relapse, incidence and severity of acute GVHD and chronic GVHD. Start time was the date of transplant for all endpoints. NRM was defined as death without relapse/progression, PFS was defined as survival without relapse or progression. Probabilities of OS and PFS were calculated using the Kaplan–Meier method. Cumulative incidence functions were used to estimate NRM and relapse incidence in a competing risk setting, death, and relapse competing with each other [8]. For the estimation of the cumulative incidence of acute and chronic GVHD, relapse and death were considered to be competing events. Multivariate analyses were performed using the Cox proportional-hazards model for all endpoints. Multivariate hazard ratios (HRs) for NRM and survival outcomes were estimated from Cox regression models. All comorbidities were put in a single multivariate model with a set of known prognostic factors. Each association of comorbidity and NRM was therefore measured independently of the presence/absence of the other comorbidities. All factors known to be potential risk factors for NRM were included in the final model: previous autologous transplantation(s), stem cell source, diagnosis, complete remission at transplant, patient age, patient gender, donor gender, intensity of conditioning, total body irradiation, in vivo T-cell depletion, CMV status, and KPS score. A stepwise AIC procedure was finally done in order to determine the optimal model for NRM. The risk factors were forced into the model and the selection was done on the comorbidities. The AIC stepwise selection was performed for the comorbidities. Five comorbidities were removed from the Cox model: cerebrovascular disease, inflammatory bowel disease, rheumatologic disease, heart valve disease, and peptic ulcer. All tests were two-sided. Statistical analyses were performed with R 3.6.2 software (R Development Core Team, Vienna, Austria) packages.

## Results

### Patient characteristics and prevalence of pre-existing comorbidities

We analyzed the association of pre-existing comorbidities with NRM in adult patients receiving a first allo-HCT between 2010 and 2018 from a matched sibling or unrelated donor for hematologic malignancy reported to the EBMT. We identified 38,760 patients fulfilling the inclusion criteria. Patient characteristics are shown in Table 1. Next, we investigated the prevalence of the specific comorbidities and found that pulmonary comorbidity (21.4%), infections (7.1%), and cardiac comorbidity (5.6%) occurred most frequently. Furthermore, we found a moderate frequency of solid tumor (5.2%), hepatic comorbidity (5.2%), diabetes (4.4%), obesity (3.9%), and psychiatric disease (3.6%). Other comorbidities occurred less frequently, including arrhythmia (2.1%), heart valve (1.6%), rheumatologic comorbidity (1.5%), cerebrovascular disease (1.4%), renal comorbidity (1.1%), inflammatory bowel disease (0.8%), and peptic ulcer (0.6%).

**Table 1:** Patient characteristics in the entire cohort (first column) and according to the hematopoietic cell transplantation comorbidity index (HCT-CI). Absolute numbers and percentages are given.

Variable		Overall (*n* = 38760)	HCT-CI = 0 (*n* = 22699)	HCT-CI = 1 or 2 (*n* = 8101)	HCT-CI ≥ 3 (*n* = 7558)
Donor cell source	Bone marrow	3358 (8.7%)	2134 (9.4)	639 (7.9)	550 (7.3%)
	Peripheral blood	35402 (91.3%)	20565 (90.6)	7462 (92.1%)	7008 (92.7%)
Diagnosis	Acute leukemia	22991 (59.3%)	13417 (59.1)	4825 (59.6%)	4509 (59.7%)
	Chronic leukemia	2427 (6.3%)	1596 (7.0)	461 (5.7%)	344 (4.6%)
	Lymphoma	5070 (13.1%)	3199 (14.1)	981 (12.1%)	857 (11.3%)
	MDS/MPN	8272 (21.3%)	4487 (19.8)	1834 (22.6%)	1848 (24.5%)
Complete remission at transplant	Yes	23791 (61.4%)	14293 (63.0)	4908 (60.6%)	4376 (57.9%)
	No	14969 (38.6%)	8406 (37.0)	3193 (39.4%)	3182 (42.1%)
Patient age (years)	Median (min–max) [IQR]	53.3 (18–83.8) [41.1–61.5]	51.1 (18–79.7) [38.2–60.2]	54.8 (18–83.8) [44–62.3]	56.9 (18–78.6) [46.8–63.6]
Patient sex	Male	22896 (59.1%)	13536 (59.6)	4922 (60.8%)	4192 (55.5%)
	Female	15864 (40.9%)	9163 (40.4)	3179 (39.2%)	3366 (44.5%)
Intensity of conditioning	Reduced-intensity	20147 (52%)	10653 (46.9)	4492 (55.4%)	4747 (62.8%)
	Myeloablative	18613 (48%)	12046 (53.1)	3609 (44.6%)	2811 (37.2%)
Conditioning contains	Yes	8976 (23.2%)	5326 (23.5)	1863 (23%)	1700 (22.5%)
Total body irradiation	No	29784 (76.8%)	17373 (76.5)	6238 (77%)	5858 (77.5%)
In vivo T-cell depletion	Yes	24852 (64.4%)	14321 (63.4)	5278 (65.4%)	4957 (65.9%)
	No	13724 (35.6%)	8255 (36.6)	2795 (34.6%)	2570 (34.1%)
	missing	184	123	28	31

*MDS* myelodysplastic syndrome, *MPN* myeloproliferative neoplasia, *IQR* interquartile range.

### Association of pre-existing comorbidities with NRM

We found that NRM at 1 year after transplant in the whole population was 15.2% [14.8–15.6] and 18.5% [18.1–18.9] at 2 years. A description of the cause for death is given in Table 2. The most frequent cause of death was relapse of the underlying malignancy (46.1%). The most frequent cause of NRM was infection (20%), closely followed by GVHD ± GVHD-related infection (18.9%). NRM in the population of patients with missing data on comorbidities was similar (data not shown).

**Table 2:** Cause of death in the entire cohort (first column) and according to the hematopoietic cell transplantation comorbidity index (HCT-CI). Absolute numbers and percentages are given.

Cause of death	Overall (*n* = 15273)	HCT-CI = 0 (*n* = 8190)	HCT-CI = 1 or 2 (*n* = 3356)	HCT-CI ≥ 3 (*n* = 3564)
Original disease	6938 (46.08%)	3820 (47.4%)	1502 (45.34%)	1551 (44.02%)
GVHD	1302 (8.65%)	704 (8.74%)	286 (8.63%)	297 (8.43%)
Infection related	3007 (19.97%)	1507 (18.7%)	683 (20.62%)	774 (21.97%)
GVHD + Infection related	1538 (10.21%)	813 (10.09%)	371 (11.2%)	343 (9.74%)
Organ toxicity	804 (5.34%)	413 (5.12%)	176 (5.31%)	206 (5.85%)
Other transplant related	432 (2.87%)	244 (3.03%)	81 (2.44%)	99 (2.81%)
Other non-transplant related	813 (5.4%)	439 (5.45%)	165 (4.98%)	200 (5.68%)
Secondary malignancy	223 (1.48%)	119 (1.48%)	49 (1.48%)	53 (1.5%)
Missing	216	131	43	41

**Fig. 1:**
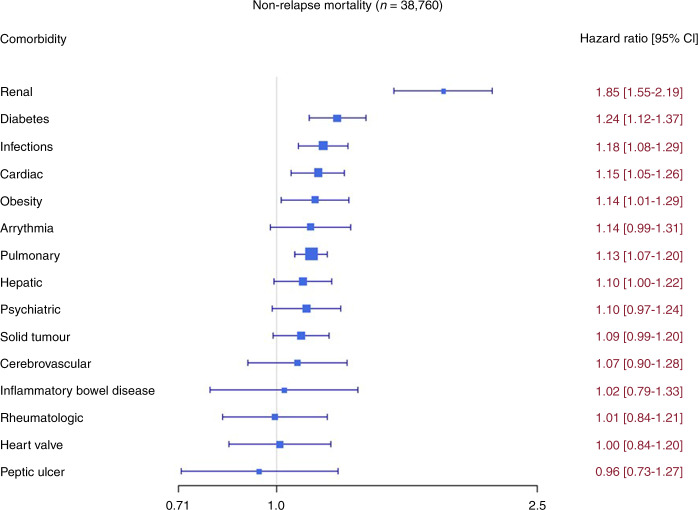
Summary of the association of pre-existing comorbidity with outcome. Shown is the association on non-relapse mortality (NRM) with frequent comorbidities.

**Fig. 2:**
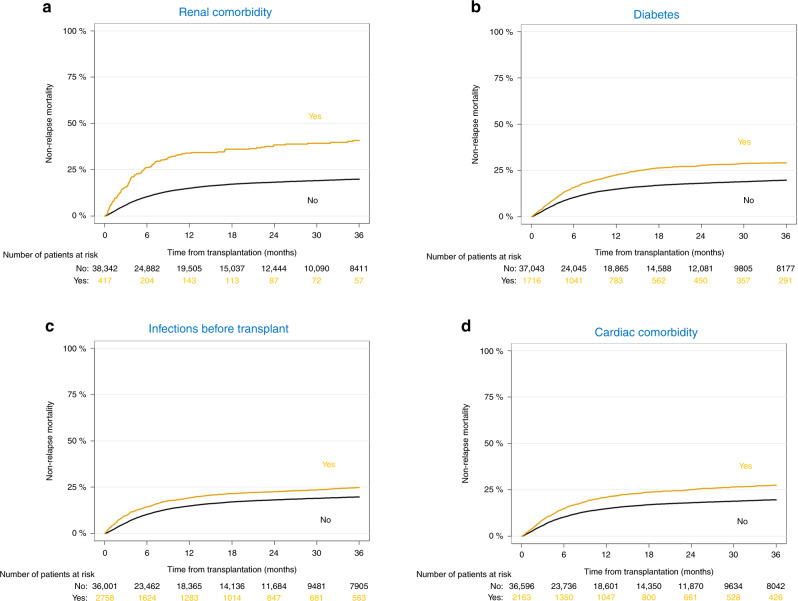
Non-relapse mortality (NRM) in univariate graphs for the comorbidities which had the highest HR for NRM in multivariate analyses. **a** Renal comorbidity, **b** diabetes, **c** infections before transplant, and **d** cardiac comorbidity.

The association of comorbidities with NRM in multivariate analysis is summarized in Fig. 1. We found that pre-existing moderate/severe renal comorbidity had a strong association with NRM (HR 1.85 [95% CI 1.55–2.19]. In addition, we found a significant association of multiple pre-existing comorbidities with NRM including diabetes (HR 1.24 [95% CI 1.12–1.37]), infections (HR 1.18 [95% CI 1.08–1.29]) cardiac comorbidity (HR 1.15 [95% CI 1.05–1.26]), obesity (HR 1.14 [95% CI 1.01–1.29]) and pulmonary comorbidity (HR 1.13 [95% CI 1.07–1.20]) However, the HR of the association of these comorbidities each with NRM was relatively low and did not exceed 1.24. Univariate outcome graphs for NRM are shown in Fig. 2 for the most frequent comorbidities which had the highest impact on NRM, including moderate/severe renal comorbidity, diabetes, infection, and cardiac comorbidity.

We then performed analyses on the association of different severities of pulmonary comorbidity as well as hepatic comorbidity on NRM. With respect to pulmonary comorbidity, 30,450 patients had no pre-existing pulmonary comorbidity, 748 had mild pulmonary comorbidity, 4328 patients had moderate pulmonary comorbidity and 2832 patients had severe pulmonary comorbidity (the grading information was missing for 402 patients). In multivariate analysis, we found that compared with no pulmonary comorbidity, the HRs of NRM in moderate and severe pulmonary comorbidity were increased with HR = 1.12 [95% CI 1.04–1.21] (*p* = 0.003) and HR = 1.33 [95% CI 1.22–1.44] (*p* < 0.0001), respectively.

With respect to hepatic comorbidity, 36,736 patients had no pre-existing hepatic comorbidity, 1584 patients had mild hepatic comorbidity, and 440 patients had moderate/severe hepatic comorbidity. In multivariate analysis, we found that the HRs of NRM in mild and moderate/severe hepatic comorbidity were increased with HR = 1.17 [95% CI 1.05–1.31] (*p* = 0.006) and HR = 1.33 [95% CI 1.08–1.62] (*p* = 0.006), respectively.

Taken together, moderate/severe renal comorbidity had a strong impact on the HR of NRM, whereas the association of other comorbidities with the HR of NRM was moderate or low.

### Association of the HCT-CI with NRM

**Fig. 3:**
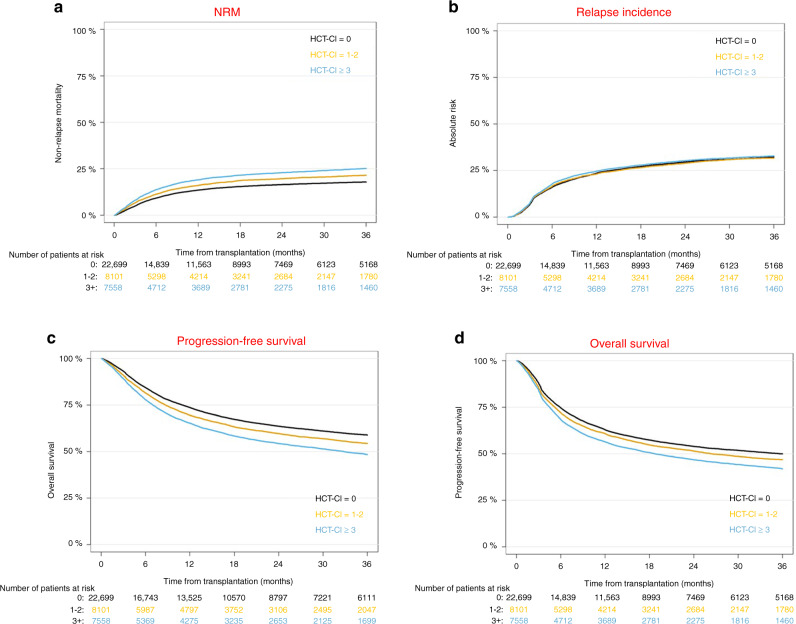
Outcome according to the hematopoietic cell transplantation comorbidity index (HCT-CI). **a** Non-relapse mortality (NRM), **b** relapse incidence, **c** progression-free survival and **d** overall survival, Shown are univariate graphs.

We first looked at the prevalence of comorbidities according to the HCT-CI definition [3, 4] and found that 22,699 (59%) patients had an HCT-CI = 0 (no comorbidities), 8101 (21%) had an HCT-CI = 1–2, and 7558 (20%) had an HCT-CI ≥ 3 (Table 1). Four hundred two patients were excluded from HCT-CI calculation, because of missing grading of pulmonary severity. The univariate association of HCT-CI with NRM is shown in Fig. 3a. NRM at one year after transplant was 13.6% [13.1–14], 16.1% [15.3–16.9] and 19% [18.1–19.9] in allo-HCT recipients with HCT-CI = 0, HCT-CI = 1–2, and HCT-CI ≥ 3, respectively. As expected, we found that patients with high HCT-CI scores preferentially received dose-reduced conditioning (RIC). The association of HCT-CI with NRM was similar in patients receiving RIC and in patients receiving myeloablative conditioning (MAC) (data not shown).

Causes of death according to HCT-CI are given in Table 2. The multivariate analyses of the association of potential risk factors with NRM are given in Table 3. We found that the risk for NRM was significantly but moderately increased in patients with HCT-CI > 0 (one or more comorbidities) vs patients with HCT-CI = 0 (no comorbidity). The HR for NRM at 1 year after transplant was 1.14 [1.07–1.21] in patients with HCT-CI = 1–2 and 1.34 [1.26–1.42] in patients with HCT-CI ≥ 3 with HCT-CI = 0 being the reference (*p* < 0.0001). In summary, we found a moderate association of the HCT-CI with HR for NRM.

**Table 3:** Multivariate analyses of risk factors potentially associated with non-relapse mortality (NRM).

Variable	Level	HR [95% CI]	*P*
HCT-CI	Reference: 0		
	1–2	1.14 [1.07–1.21]	<0.0001
	3+	1.34 [1.26–1.42]	<0.0001
Previous autologous transplantation(s)	Reference: Yes		
	No	0.85 [0.76–0.94]	0.002
Year of transplantation (5 years increment)		0.84 [0.79–0.88]	<0.0001
Cell source	Reference: bone marrow		
	Peripheral blood	0.99 [0.9–1.08]	0.82
Type of donor	Reference: Identical sibling		
	Unrelated	1.47 [1.38–1.56]	<0.0001
Diagnosis	Reference: acute leukemia		
	Chronic leukemia	0.89 [0.8–0.99]	0.033
	Lymphoma	1.17 [1.06–1.29]	0.001
	MDS/MPN	1 [0.94–1.08]	0.89
Complete remission at transplant	Reference: yes		
	No	1.34 [1.26–1.42]	<0.0001
Patient age (5 years increment)		1.13 [1.12–1.14]	<0.0001
Patient sex	Reference: male		
	Female	0.88 [0.83–0.92]	<0.0001
Donor sex	Reference: male		
	Female	1.17 [1.11–1.23]	<0.0001
Patient CMV	Reference: positive		
	Negative	0.81 [0.77–0.86]	<0.0001
Donor CMV	Reference: Positive		
	Negative	1.01 [0.96–1.07]	0.59
Karnofsky score	Reference: <80		
	≥80	0.57 [0.52–0.62]	<0.0001
Intensity of conditioning	Reference: RIC		
	MAC	1.15 [1.09–1.22]	<0.0001
TBI (all dosages)	Reference: yes		
	No	0.96 [0.9–1.02]	0.17
In vivo T-cell depletion	Reference: ATG/Campath		
	No	1.1 [1.04–1.17]	0.0009

*CMV* cytomegaly virus, *TBI* total body irradiation, *ATG* anti-thymocyte globulin, *MDS* myelodysplastic syndrome, *MPN* myeloproliferative neoplasia.

### Secondary outcome variables

The results of the univariate analyses of secondary study endpoints including relapse incidence, PFS, OS, incidence, and severity of acute GVHD and chronic GVHD are given in Table 4. Figure 3b–d shows the univariate association of HCT-CI with relapse incidence, PFS, and OS. In multivariate analyses, we found that the HR for relapse incidence at 1 year after transplant was 0.99 [95% CI 0.95–1.05] in patients with HCT-CI = 1–2 and 1.07 [95% CI 1.02–1.13] in patients with HCT-CI ≥ 3, with HCT-CI = 0 being the referent group (*p* < 0.0001). The HR for PFS was 1.05 [95% CI 1.01–1.09] in patients with HCT-CI = 1–2, and 1.17 [95% CI 1.13-1.22] in patients with HCT-CI ≥ 3. The respective HRs for OS were 1.09 [95% CI 1.04–1.14] in patients with HCT-CI = 1–2 and 1.26 [95% CI 1.21–1.32] in patients with HCT-CI ≥ 3. The HR for acute GVHD grades III-IV was 1.03 [95% CI 0.95–1.12] in patients with HCT-CI = 1–2 and 1.14 [95% CI 1.05–1.24] in patients with HCT-CI ≥ 3. The HR for extensive chronic GVHD was 1.13 [95% CI 1.06–1.2] in patients with HCT-CI = 1–2 and 1.15 [95% CI 1.07–1.23] in patients with HCT-CI ≥ 3. In summary, we found that the association of comorbidities, (as defined by the HCT-CI), with secondary outcome variables was moderate or low.

**Table 4:** Univariate analyses of outcome variables in the entire cohort (first column) and according to the hematopoietic cell transplantation comorbidity index (HCT-CI). Absolute numbers and percentages are given.

Outcome variable	Time	Entire cohort (*n* = 38760)	HCT-CI = 0 (*n* = 22,699)	HCT-CI = 1 or 2 (*n* = 8101)	HCT-CI ≥ 3 (*n* = 7558)	*P*
Non-relapse mortality	At 6 months [95% CI]	10.6% [10.3–10.9]	9.2% [8.9–9.6]	11.4% [10.7–12.1]	13.8% [13–14.6]	<0.0001
	At 12 months [95% CI]	15.2% [14.8–15.6]	13.6% [13.1–14]	16.1% [15.3–16.9]	19% [18.1–19.9]	
	At 24 months [95% CI]	18.5% [18.1–18.9]	16.5% [16–17]	19.7% [18.7–20.6]	22.9% [21.9–23.9]	
Overall survival	At 6 months [95% CI]	82.4% [82–82.8]	84.4% [83.9-84.9]	81.4% [80.6–82.3]	77.8% [76.9–78.8]	<0.0001
	At 12 months [95% CI]	71% [70.6–71.5]	73.6% [73–74.2]	69.5% [68.4–70.5]	65.3% [64.2–66.4]	
	At 24 months [95% CI]	60.8% [60.3–61.4]	63.6% [62.9–64.3]	59.6% [58.5–60.8]	54.2% [53–55.5]	
Progression-free survival	At 6 months [95% CI]	72.8% [72.3–73.2]	74.6% [74–75.2]	71.9% [70.9-72.9]	68.3% [67.2–69.4]	<0.0001
	At 12 months [95% CI]	61.1% [60.6–61.6]	62.8% [62.2–63.5]	60.8% [59.7–61.9]	56.3% [55.2–57.5]	
	At 24 months [95% CI]	51.9% [51.4–52.5]	53.9% [53.2–54.7]	51.4% [50.2–52.6]	46.8% [45.6–48]	
Relapse incidence	At 6 months [95% CI]	16.6% [16.3–17]	16.1% [15.6–16.6]	16.8% [15.9–17.6]	17.9% [17–18.8]	0.25
	At 12 months [95% CI]	23.7% [23.3–24.2]	23.6% [23–24.2]	23.1% [22.2–24.1]	24.7% [23.7–25.7]	
	At 24 months [95% CI]	29.6% [29.1–30.1]	29.6% [28.9–30.2]	28.9% [27.9–30]	30.3% [29.2–31.4]	
Acute grade II-IV GVHD	At 30 days [95% CI]	16.1% [15.8–16.5]	15.8% [15.3–16.3]	16.5% [15.7–17.3]	16.7% [15.9–17.6]	0.002
	At 100 days [95% CI]	27.5% [27.1–28]	27% [26.4–27.6]	27.5% [26.5–28.5]	29.1% [28.1–30.2]	
	At 180 days [95% CI]	28.2% [27.8–28.7]	27.7% [27.1–28.3]	28.2% [27.2–29.2]	29.7% [28.7–30.8]	
Acute grade III-IV GVHD	At 30 days [95% CI]	5% [4.8–5.2]	4.7% [4.5–5]	5.3% [4.8–5.8]	5.4% [4.9–6]	0.009
	At 100 days [95% CI]	10.6% [10.3–10.9]	10.3% [9.9–10.7]	10.5% [9.8–11.2]	11.6% [10.9–12.4]	
	At 180 days [95% CI]	10.9% [10.6–11.3]	10.6% [10.2–11.1]	10.8% [10.1–11.5]	12% [11.2–12.7]	
Chronic GVHD	At 6 months [95% CI]	18.9% [18.5–19.4]	19.4% [18.8–20]	18.7% [17.8–19.6]	17.8% [16.9–18.7]	0.006
	At 12 months [95% CI]	30.6% [30.1–31.1]	31.1% [30.5–31.8]	30.2% [29.1–31.3]	29.4% [28.3–30.4]	
	At 24 months [95% CI]	36.1% [35.6–36.6]	36.7% [36–37.4]	35.8% [34.6–36.9]	34.8% [33.6–35.9]	
Extensive chronic GVHD	At 6 months [95% CI]	7.6% [7.3–7.9]	7.6% [7.2–8]	7.7% [7.1–8.4]	7.4% [6.8–8.1]	0.006
	At 12 months [95% CI]	12.7% [12.3–13.1]	12.5% [12–13]	13% [12.3–13.8]	12.9% [12.1–13.7]	
	At 24 months [95% CI]	16.2% [15.8–16.6]	15.7% [15.2–16.2]	17.2% [16.3–18.1]	16.6% [15.7–17.5]	

## Discussion

The number of comorbidities increases with age making comorbidities a significant concern in the aging population of allo-HCT recipients. Nowadays we are more likely to encounter patients with multiple comorbidities that can influence transplant‐related mortality. The current analysis derives from a large and recent data set, which is representative for the overall allo-HCT activity in patients with hematologic malignancies in Europe. Our results demonstrate that there is a high prevalence of comorbidities in the allo-HCT population. We found that moderate/severe renal comorbidity is strongly associated with an increased HR for NRM. However, the HRs for NRM were only moderately increased in multivariate analyses for other comorbidities including severe pulmonary comorbidity. Our results confirm data from a single center analysis that already has pointed in the same direction. The authors found that renal comorbidity defined by an estimated glomerular filtration rate <60 mL/min/1.73 m^2^ was associated to an increased HR for NRM, whereas other comorbidities had more moderate impact [9].

When putting the present results in perspective with the previous data from North America, which was the basis for the HCT-CI calculation, the impact of non-renal comorbidities on NRM is much lower in the current EBMT population [3, 4]. There are some differences in the prevalence of comorbidities in both studies, but also many similarities—e.g., that pulmonary comorbidity occurred most frequently. Since we used the same definitions for comorbidities that were used in the HCT-CI calculation cohort, we consider the data presented here to be significant and valid as a comparator. When looking at the HRs of NRM of severe pulmonary and hepatic comorbidities, it becomes clear that the NRM-associated risk is considerably lower in the more recent EBMT population. In the HCT-CI calculation, the HR for severe pulmonary comorbidity was 3.7 and for moderate/severe hepatic comorbidity was 3.9. In the current study, we found respective HRs of 1.3 of NRM associated with both severe pulmonary and moderate/severe hepatic comorbidities. Our study design does not allow analysis of the reasons for this significant difference of association of pre-existing comorbidity. However, we believe that three main factors are most likely to be the cause of the reduced impact of comorbidities in the more recent cohort: (1) differences in patient characteristics as well as treatment regimens; (2) improved management of allo-HCT-related complications in more recent times; and (3) better awareness and patient selection guided by the presence of comorbidities.

The HCT-CI was originally calculated from comorbidity and NRM data of patients undergoing allo-HCT between 1997 and 2003 [3] and then tested prospectively in a population undergoing allo-HCT between 2007 and 2009 [10]. When comparing the characteristics of the original HCT-CI population and of the 2015 publication with our current cohort it is evident that our transplantation strategies have changed over time. The recent EBMT cohort contains a higher frequency of dose-reduced conditioning (52% vs. 28% original HCT-CI cohort), unrelated donors (64% vs. 42%), and older patients (median age 53.3 vs. 44.8 years) as compared to the original HCT-CI cohort. Over the last few decades, considerable progress has been made regarding the reduction of NRM, which is most likely due to progress in intensive care medicine, as well as to improved management of GVHD, conditioning regimens, and prevention/treatment of infectious complications in alloSCT recipients [5, 9, 11–13].

We hypothesize that probably because of the above-mentioned favorable developments, the HCT-CI is not a strong predictor of the HR of NRM in the current EBMT population. Our data validate the recent results from a single center analysis that compared eight different scores, which are in routine use to predict mortality after allo-HCT [14]. Of note, the predictive value of HCT-CI of NRM was lower as compared with the other seven scores. Three of the scores that include biomarkers or disease-specific data (Revised Pre-transplantation Assessment of Mortality [rPAM], Revised Disease Risk Index [rDRI] and Endothelial Activation and Stress Index [EASIX]) performed best in predicting NRM after allo-HCT, although no score had optimal predictive value [15–17]. It is likely that the relatively low association of HCT-CI with HR of NRM in this study is due to its exclusive focus on pre-existing comorbidities [9, 14].

In summary, we provide favorable data demonstrating that the negative impact of pre-existing comorbidities on NRM is not as strong in recent times as originally calculated. There are several implications of our study for current management and for the future perspective: (A) Although the impact of comorbidities on NRM has decreased, it remains vital to thoroughly evaluate allo-HCT patients for comorbidities and to critically consider transplant indications in patients with severe comorbid conditions; (B) Patients with severe hepatic or pulmonary comorbidities according the definitions used by the EBMT and by HCT-CI should not be excluded per se from allo-HCT. However, our study does not allow a subgroup analysis of patients with impaired FEF1 between 50 and 65%. Furthermore, we do not have the information if pulmonary functional test data were available for each patient and if these data were corrected for the degree of anemia in all cases. Further studies are needed to predict the outcome in this subgroup of patients with severe pulmonary comorbidity; (C) The most adequate prediction of NRM and overall mortality after allo-HCT can probably be assessed by combining scores that take into consideration comorbidities, disease-specific information, age, and biomarkers. Analyses of the predictive value of those combined clinical data/biomarker scores in current and representative data sets are currently missing and are warranted in the future.
